# Effectiveness of Expressed Breast Milk Mouthwash for Infant Oral Hygiene

**DOI:** 10.3390/nursrep16060195

**Published:** 2026-06-08

**Authors:** Reda Elsahy, Thaer Momani

**Affiliations:** 1 Nursing Department, Al Jalila Children’s Hospital, Dubai Health, Dubai P.O. Box 2556, United Arab Emirates; 2 Hind Bint Maktoum College of Nursing and Midwifery, Mohammed Bin Rashid University of Medicine and Health Sciences, Dubai Health, Dubai P.O. Box 505055, United Arab Emirates; thaer.almomani@dubaihealth.ae

**Keywords:** expressed breast milk, chlorhexidine, infant oral hygiene, oropharyngeal colostrum administration, nursing, ventilator-associated pneumonia, preterm infants

## Abstract

**Background/Objectives:** Maintaining oral hygiene in infants in neonatal and pediatric intensive care is essential for preventing ventilator-associated pneumonia (VAP). Chlorhexidine (CHX) is widely used in adults but its safety and efficacy in infants remain uncertain, and it is not recommended for children under 6 years due to rinsing difficulties and mucosal irritation risk. Expressed breast milk (EBM), rich in immunological and antimicrobial components, has been explored as a biologically appropriate alternative. This review synthesizes evidence on EBM effectiveness and safety and contextualizes it against limited indirect evidence for CHX, as no head-to-head comparative trials were identified. **Methods:** A systematic search of PubMed, EMBASE, Cochrane Library, CINAHL, and Web of Science (January 2015–January 2026) identified randomized and non-randomized studies involving infants ≤ 12 months receiving EBM, colostrum, or CHX for oral care. Risk of bias was assessed using RoB 2 for RCTs and ROBINS-I for non-RCTs. Due to substantial clinical and methodological heterogeneity (differing populations, dosages, frequencies, delivery methods, and outcome definitions), a narrative synthesis was performed. **Results:** Seventeen studies met inclusion criteria (11 RCTs, n = 1185; 6 non-RCTs, n > 3000). EBM and oropharyngeal colostrum were associated with trends toward lower VAP incidence trends (0–4%), reduced bacterial colonization, improved oral health indices, shorter mechanical ventilation time, and reduced ICU/hospital stays, with no reported adverse events. Evidence for CHX in infants was limited to a single paediatric RCT and bundled interventions, showing no significant VAP reduction and associations with mucosal irritation. The risk of bias was generally low to moderate. **Conclusions:** Indirect evidence suggests EBM is a potentially beneficial option for infant oral hygiene, with favourable trends for infection-related outcomes and recovery parameters. However, all EBM–CHX comparisons are indirect, and CHX evidence in infants is limited by the risk of bias and heterogeneity. High-quality head-to-head randomized controlled trials are needed to determine optimal strategies and inform guidelines.

## 1. Introduction

Effective oral hygiene for infants, particularly those admitted to neonatal and paediatric intensive care units (ICUs), is essential for preventing healthcare-associated infections such as VAP. The oral cavity serves as a primary reservoir for respiratory pathogens, and in high-risk infants, including preterm neonates and those requiring mechanical ventilation, poor oral hygiene contributes to increased microbial colonization, illness severity, prolonged hospitalization, and higher morbidity [1,2,3]. VAP remains one of the most common ICU-acquired infections in children and is closely linked to deteriorated oral health and pathogenic colonization [2,3].

Current oral care practices in paediatric ICUs typically include mechanical cleansing and, in some cases, antiseptic solutions. Although CHX is widely used in adult practice due to its broad antimicrobial activity by cationic antiseptic that disrupts bacterial cell membranes [4], its suitability for infants is increasingly questioned. CHX is not recommended for children under 6 years of age, largely due to their inability to rinse effectively and the risk of mucosal irritation, ulceration, tooth staining, taste alteration, and other adverse effects [5,6]. Paediatric studies have shown no significant reduction in VAP incidence with 0.12% CHX [7], and adult data have raised additional concerns regarding potential systemic risks, including increased odds of sepsis and mortality without corresponding reductions in pneumonia [8,9]. These findings underscore the uncertainty surrounding CHX’s safety and effectiveness in vulnerable infant populations.

Expressed Breast Milk (EBM) has emerged as a biologically appropriate and physiologically compatible option for infant oral care. EBM contains multiple bioactive components, including secretory immunoglobulin A (sIgA), lactoferrin, lysozymes, growth factors, and beneficial microbiota, that support mucosal immunity, reduce pathogen adherence, and promote epithelial integrity [10,11]. Preliminary clinical evidence suggests that EBM may lower oral bacterial load, enhance oral mucosal healing, improve microbial diversity, and reduce infection-related outcomes such as VAP and sepsis, without the adverse effects associated with chemical antiseptics [12,13]. Oropharyngeal administration of colostrum has also demonstrated benefits in accelerating feeding tolerance and reducing inflammatory markers. However, significant variability exists in dosing regimens, delivery methods (e.g., swabbing, drops, syringe rinsing), frequency of application, and duration of interventions, limiting the comparability of available studies [7,14].

Despite growing interest in EBM for infant oral hygiene, no head-to-head randomized controlled trials (RCTs) directly comparing EBM with CHX in infants have been conducted. Available evidence therefore relies on indirect comparisons, derived from separate studies evaluating each intervention against placebo, saline, or standard care [8,15]. This limitation complicates direct conclusions about comparative effectiveness and highlights the need for systematic synthesis of the current evidence base.

This systematic review aims to evaluate the effectiveness and safety of EBM (including oropharyngeal colostrum) for oral hygiene in infants in healthcare settings. The review assesses key clinical outcomes including VAP incidence, bacterial colonization, oral health indices, adverse effects, mechanical ventilation time (MVT), length of hospital stay (LOS), and necrotizing enterocolitis (NEC). By synthesizing heterogeneous evidence across neonatal and paediatric care settings, this review seeks to inform evidence-based nursing practice and identify priorities for future research, including the need for high-quality, head-to-head RCTs to guide optimal oral care strategies for infants.

## 2. Materials and Methods

This systematic review was conducted in accordance with the Preferred Reporting Items for Systematic Reviews and Meta-Analyses (PRISMA) 2020 guidelines [16]. The review protocol was registered in PROSPERO (Registration ID: CRD420261290246), and designed to identify, evaluate, and synthesize clinical and microbiological evidence regarding the effectiveness and safety of EBM, including oropharyngeal colostrum, for infant oral hygiene in healthcare settings. A secondary objective was to contextualize these findings against the limited indirect evidence available for CHX, as no direct head-to-head comparative trials were identified during the preliminary scoping phase.

### 2.1. Search Strategy and Selection Process

A comprehensive systematic search was performed across five electronic databases: PubMed, EMBASE, Cochrane Library, CINAHL, and Web of Science, for studies published between January 2015 and January 2026. Due to resource limitations, the search was restricted to English-language publications. The search strategy utilized a combination of Medical Subject Headings (MeSH) and free-text terms such as “expressed breast milk,” “oropharyngeal colostrum,” “chlorhexidine mouthwash,” “infant oral hygiene,” “ventilator-associated pneumonia,” and “oral immune therapy.” Boolean operators (AND/OR) were applied to maximize search sensitivity, and no geographic restrictions were imposed. To ensure literature saturation, the reference lists of all included studies and relevant review articles were manually screened. Duplicate records were identified and removed using Endnote (Clarivate, Philadelphia, PA, USA) and Covidence software (Veritas Health Innovation, Melbourne, Australia; accessed on 15 March 2026). Two reviewers independently screened titles and abstracts against the eligibility criteria, followed by a double-blind full-text assessment. Any discrepancies during the selection process were resolved through formal discussion.

### 2.2. Study Selection

The review followed a structured PICOS framework. Two reviewers independently assessed studies against the eligibility criteria. Discrepancies were resolved through formal discussion, and a third reviewer was available for consultation when required.

Inclusion Criteria

(1) Population (P): Infants (term or preterm) aged 12 months or younger receiving oral care within neonatal or paediatric intensive care units.
(2) Intervention (I): Oral care using EBM, mother’s own milk, or colostrum, administered via swabbing, drops, or oropharyngeal application.
(3) Comparison (C): Placebo or control treatments such as sterile water, normal saline, sodium bicarbonate, chlorhexidine, or standard institutional oral care protocols.
(4) Outcome (O): Primary outcomes: Incidence of VAP and Bacterial colonization patterns such as *Streptococcus mutans*, *Candida* spp., and *Klebsiella organisms*. Secondary outcomes: Oral health indices, MVT, LOS, NEC, Late-onset sepsis, Adverse effects (e.g., mucosal irritation), and Caregiver acceptability
(5) Study Design (S): RCTs, non-RCTs, and quasi-experimental studies.

Exclusion Criteria

(1) Studies involving children older than 12 months.
(2) Studies on infants with immune diseases or those receiving immunosuppressive therapy.
(3) Studies in which complete data could not be obtained.
(4) Retrospective studies, reviews, systematic reviews, case reports, letters, conference abstracts, or editorials.

### 2.3. Data Extraction

Data were extracted using a standardized template. Extracted information included: (1) Study characteristics (design, setting, publication year). (2) Infant demographics, including gestational age, birth weight, and clinical status. (3) Intervention details: dosage, frequency, mode of delivery (swabbing, drops, or oropharyngeal application). (4) Comparator characteristics, including placebo (sterile water or saline), sodium bicarbonate, CHX, or institutional standard care. (5) Primary outcomes: incidence of VAP and patterns of bacterial colonization (e.g., *Streptococcus mutans*, *Candida* spp., *Klebsiella*). (6) Secondary outcomes: oral health indices, MVT, LOS, NEC, late-onset sepsis, adverse effects (e.g., mucosal irritation), and caregiver acceptability. (7) Additional protocol details, such as duration of therapy and timing of measurements.

### 2.4. Quality Assessment

The quality of the included studies was rigorously evaluated using domain-specific assessment tools. Two reviewers performed the assessments independently, and any disagreements were resolved through formal discussion.

**Figure 1:**
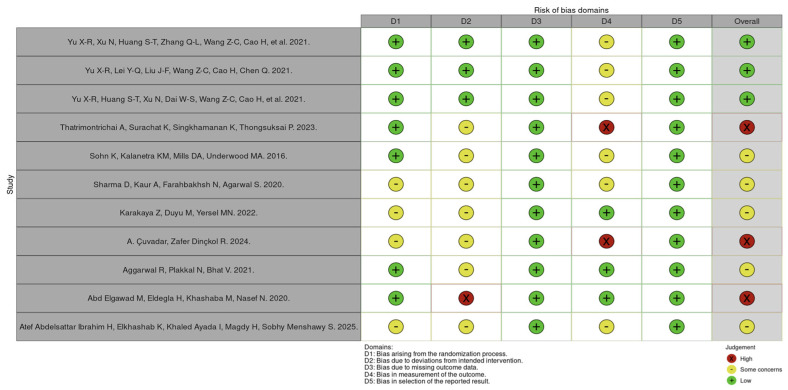
Risk of bias assessment (RoB 2) for the 11 included RCTs [7,12,13,15,18,19,20,21,22,23,24].

Randomized Controlled Trials (RCTs) were assessed using the Cochrane Risk of Bias 2.0 (RoB 2) tool [17], which evaluates five domains: bias arising from the randomization process, deviations from intended interventions, missing outcome data, measurement of the outcome, and selection of the reported result. Of the 11 RCTs, three studies (all by Yu et al., 2021) were rated low risk of bias across all domains [15,18,19]. Five studies (Sohn et al. 2016 [13], Sharma et al. 2020 [20], Karakaya et al. 2022 [21], Aggarwal et al. 2021 [22], and A. Ibrahim et al. 2025 [7]) were rated as having some concerns, primarily in randomization and deviations from intended interventions. The remaining three studies (Thatrimontrichai et al. 2023 [23], Çuvadar et al. 2024 [24], and Abd-Elgawad et al. 2020 [12]) were rated high risk of bias, driven mainly by high risk in measurement of the outcome and deviations from intended interventions. Overall, the RCT evidence base showed generally low to moderate risk of bias, with the most common concerns concentrated in outcome assessment and blinding of caregivers delivering the intervention (Figure 1).

**Figure 2:**
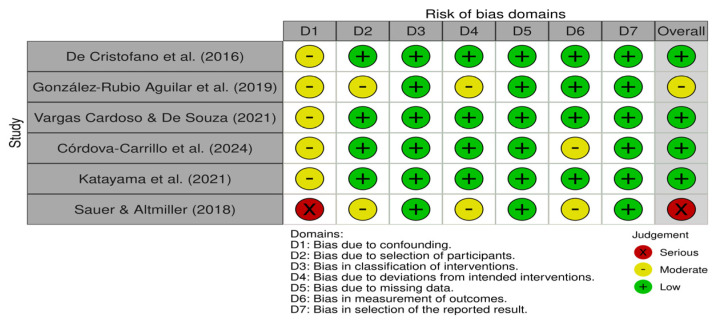
Risk of bias assessment using ROBINS-I for the 6 included non-RCTs [8,9,26,27,28,29].

Non-randomized studies were evaluated using the ROBINS-I tool (https://www.bristol.ac.uk/population-health-sciences/centres/cresyda/barr/ (accessed on 26 March 2026)) [25], which examines seven domains: confounding, selection of participants, classification of interventions, deviations from intended interventions, missing data, measurement of outcomes, and selection of the reported result. Of the six non-RCTs, four (De Cristofano et al. 2016 [26], Vargas Cardoso & De Souza 2021 [27], Córdova-Carrillo et al. 2024 [28], and Katayama et al. 2021 [8]) were judged low overall risk of bias. One study (González-Rubio Aguilar et al. 2019 [29]) was rated moderate risk, primarily due to confounding and selection of participants. One study (Sauer & Altmiller 2018 [9]) was rated serious risk of bias, driven by serious confounding and moderate concerns in participant selection and outcome measurement. Domain-level synthesis showed that the main vulnerabilities across non-RCTs were confounding (D1), reflecting design-level limitations (Figure 2).

Overall, the risk of bias across the 17 included studies was judged to be low to moderate, with localized high or serious risk in a minority of studies, primarily related to outcome measurement (RCTs) and confounding (non-RCTs).

### 2.5. Data Synthesis and Heterogeneity

A meta-analysis was deemed inappropriate due to substantial clinical and methodological heterogeneity across the included studies. This heterogeneity stemmed from variations in patient populations (ranging from extremely preterm neonates to postoperative paediatric cardiac patients), inconsistent definitions of VAP and other outcomes, diverse EBM administration protocols (dosages, frequencies, durations, delivery methods), and differences in comparator groups. Furthermore, the absence of direct head-to-head trials comparing EBM and CHX necessitated a narrative synthesis approach. Results were summarized through thematic analysis of the 11 RCTs (n = 1185) and 6 non-RCTs (n > 3000), utilizing structured evidence tables to describe patterns in clinical effectiveness and safety profiles. This narrative synthesis specifically integrated the risk-of-bias assessments into the interpretation of findings to ensure a cautious and evidence-based conclusion regarding the utility of EBM in infant populations.

## 3. Results

### 3.1. Study Selection

**Figure 3:**
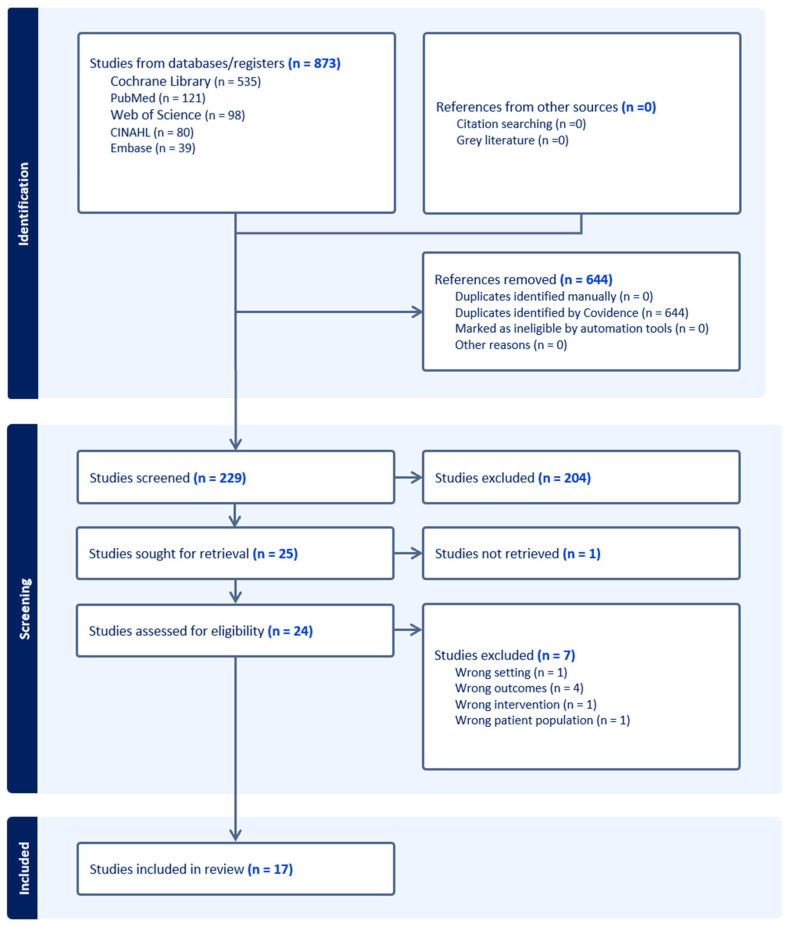
PRISMA flowchart of study selection.

The PRISMA flow diagram (Figure 3) illustrates the study selection process. A total of 873 records were identified across five databases (Cochrane Library n = 535, PubMed n = 121, Web of Science n = 98, CINAHL n = 80, EMBASE n = 39). After removal of 644 duplicates using Covidence software, 229 records underwent title and abstract screening, of which 204 were excluded. One full-text article could not be retrieved, leaving 24 for eligibility assessment. Seven were excluded due to wrong setting (n = 1), wrong outcomes (n = 4), wrong intervention (n = 1), or wrong population (n = 1). Ultimately, 17 studies were included: 11 RCTs involving 1185 infants and 6 non-RCT or quasi-experimental studies involving more than 3000 participants.

### 3.2. Study Characteristics

The studies were included: 11 RCTs (n = 1185 participants) and 6 non-RCTs (n > 3000 participants). Studies were primarily conducted in Asia (12/17), with settings in NICUs (14/17) or PICUs (3/17). Participants primarily included term and preterm infants aged 0–12 months, very low-birthweight (VLBW) preterm infants (gestational age 26–31 weeks, birth weight 850–1670 g), and infants undergoing cardiac surgery requiring mechanical ventilation (weight approximately 4 kg). Interventions focused on EBM, colostrum, or mother’s own milk (0.1–2 mL, q3–8 h) via swabbing, drops, or rinsing, as well as 0.12% CHX mouthwash or rinses in CHX studies, versus comparators like sterile water, saline, or sodium bicarbonate. Outcomes included VAP (CC criteria in 8 studies), bacterial colonization (culture in 6 studies), oral health indices (Newborn Oral Health Assessment Tool (NOHAT) in 3 studies), MVT, LOS, NEC (Bell staging in 5 studies), and sepsis (culture-proven in 7 studies). See Table 1 and Table 2 for details.

### 3.3. Impact on Ventilator-Associated Pneumonia (VAP)

Across multiple settings, EBM and colostrum were associated with trends toward lower infection rates, including VAP and postoperative pneumonia, although findings should be interpreted cautiously due to moderate-to-high risk of bias and heterogeneity in outcome definitions. In a three-arm RCT among post-cardiac surgery infants, EBM was associated with a trend toward lower postoperative pneumonia rates (3.2%) compared with physiological saline (22.6%) and sodium bicarbonate (19.4%; *p* = 0.031) [18]. In infants undergoing ventricular septal defect repair, pneumonia incidence showed a trend toward reduction from 21.4% (saline) to 3.6% with EBM (*p* = 0.043) [19]. Another cardiothoracic surgery RCT demonstrated favorable trends toward reductions in thrush and VAP infections with EBM compared to sodium bicarbonate (*p* < 0.05) [18]. Among preterm infants, oropharyngeal administration of mother’s milk (OPAMM) was associated with trends toward reduced VAP from 11% (control) to 3% (*p* = 0.049) [12]. Colostrum administration in NICUs showed no significant difference in composite outcomes (LOS/NEC/death; *p* = 0.50) or VAP incidence [22]. A more recent trial found significantly reduced sepsis rates with prolonged colostrum administration (*p* < 0.001) [23].

In contrast, evidence for CHX in infants was limited to a single pediatric RCT and bundled interventions. A PICU RCT (n = 174) found no difference in VAP incidence between 0.12% CHX and saline (29.5 vs. 35.1 per 1000 ventilator-days; *p* = 0.63) [21]. Bundled oral care interventions including CHX reduced VAP rates over time, but the individual CHX contribution could not be isolated [27]. In a large surgical cohort (n = 2535), professional toothbrushing reduced pneumonia risk (OR 0.06, *p* = 0.02), but adding a CHX spray provided no additional benefit (OR 0.87, *p* = 0.59) [29].

### 3.4. Bacterial Colonization and Oral Health

Evidence showed supportive trends for EBM in improving oral microbial balance and mucosal health in several studies, though findings are limited by moderate-to-high risk of bias and variability in outcome measures. Oropharyngeal MOM was linked to preservation of beneficial taxa such as *Bifidobacterium bifidum* and *Faecalibacterium* while reducing sepsis risk (RR 0.64) [13]. In one RCT, EBM was associated with significantly improved oral health (NOHAT) scores over 10 days, promoting faster mucosal healing than distilled water (*p* < 0.05) [24]. In non-RCTs, sterile water swabbing decreased oral bacterial load (*p* = 0.009) [8], while retrospective colostrum swabbing eliminated central line-associated bloodstream infection (CLABSI) events in a small preterm cohort [9]. Exclusive breastfeeding was associated with markedly lower *Streptococcus mutans* counts (9 × 10^3^ CFU/mL) compared with formula feeding (78 × 10^3^ CFU/mL; *p* = 0.04) [23]. Direct CHX-only colonization data in infants were limited; improvements reported in bundled interventions were not attributable solely to CHX [27].

### 3.5. Clinical Recovery Metrics

EBM use was associated with trends toward accelerated recovery and reduced resource utilization in several studies, although heterogeneity in populations and protocols limits generalizability. Reduced MVT was observed (2.8 ± 1.5 days vs. 4.2 ± 2.0 days with saline; *p* = 0.029) [19]. Shorter ICU stay was reported (5.8 ± 1.8 days vs. 7.8 ± 2.7 days; *p* = 0.028) [18]. Lower inflammatory markers were found (C-reactive protein (CRP) 3.4 ± 1.2 mg/L vs. 5.8 ± 1.6 mg/L with sodium bicarbonate; *p* < 0.001) [18]. Faster transition to full enteral feeding was observed (11.1 vs. 15.6 days; *p* < 0.01) [12]. CHX interventions did not demonstrate consistent improvements in MVT, LOS, or recovery indicators (*p* > 0.05) [21].

### 3.6. Safety and Secondary Morbidities

EBM and colostrum were consistently reported as safe, with no aspiration events, hemodynamic instability, or adverse reactions across all trials. In contrast, CHX was associated with mucosal irritation, oral discomfort, staining, and taste alteration [9,21]. Regarding secondary outcomes, colostrum administration did not increase NEC risk (*p* = 0.50) [20,22]. Several studies reported shorter hospital stays and reduced sepsis incidence with prolonged colostrum protocols [12,13,23]. No study demonstrated increased mortality with EBM or colostrum interventions.

### 3.7. GRADE

GRADE ratings for primary outcomes: VAP reduction with EBM was moderate (downgraded for RoB in blinding and inconsistency in protocols); bacterial colonization was low (downgraded for RoB and imprecision in small samples). For CHX, VAP evidence was low (downgraded for indirectness and limited studies). See Table 3 for details.

## 4. Discussion

The synthesized evidence from 17 studies suggests that EBM, including oropharyngeal colostrum, is a safe and potentially beneficial option for infant oral hygiene in neonatal and paediatric intensive care settings. Across the included RCTs and non-RCTs, EBM was associated with favourable patterns in infection-related outcomes (e.g., lower VAP and sepsis rates), improved oral health indices, shorter MVT and LOS, and accelerated enteral feeding, with no reported adverse events [12,15,18,19,22]. These associations align with EBM’s immunological and antimicrobial properties, including lactoferrin, secretory immunoglobulin A (sIgA), and lysozymes, which may support mucosal integrity, modulate microbiota balance, and reduce pathogen adherence without the local irritation risks of chemical antiseptics [10,11,13].

In NICUs, oropharyngeal administration of colostrum or MOM was linked to trends toward lower infection rates and improved feeding tolerance. For instant, a study reported comparable composite outcomes for late-onset sepsis, NEC, and death (*p* = 0.50) but noted trends toward reduced VAP and faster enteral feeding [22]. Another observed a borderline reduction in VAP (3% vs. 11%; *p* = 0.049) and shorter time to full feeds (*p* < 0.01), potentially attributable to EBM’s immunomodulatory effects on the oropharyngeal mucosa [12]. Additional evidence indicated trends toward improved microbiota diversity and lower sepsis risk (RR 0.62), with preservation of beneficial taxa such as *Bifidobacterium bifidum* [23]. Non-RCT data were consistent with these patterns, showing reduced early VAP incidence and elimination of central line-associated bloodstream infections (CLABSIs) in small cohorts [8,9]. However, these findings should be interpreted cautiously given moderate-to-high risk of bias, particularly from confounding and selection bias in non-RCT designs, and substantial heterogeneity in protocols and outcome definitions.

In postoperative settings, particularly among infants undergoing cardiac surgery, EBM was associated with lower rates of postoperative pneumonia and inflammatory markers (e.g., CRP; *p* < 0.001) compared to saline or sodium bicarbonate in multiple RCTs [15,18,19]. These patterns suggest potential benefits in mitigating postoperative complications, with shorter MVT (*p* = 0.029) and ICU LOS (*p* = 0.028) observed [18,19]. Improved Newborn Oral Health Assessment Tool (NOHAT) scores and faster mucosal healing was also reported [24]. Quasi-experimental studies incorporating oral hygiene bundles showed reductions in pneumonia incidence and VAP rates, but individual contributions of CHX were unclear and often negligible [26,27,29].

Evidence regarding CHX in infants remains limited and indirect. A single paediatric RCT found no significant reduction in VAP incidence with 0.12% CHX compared to placebo (*p* = 0.63) [21], and CHX may be associated with mucosal irritation and other local side effects [6,21]. While CHX is effective in adult populations, its application in infants is constrained by age-related limitations (e.g., inability to rinse) and the absence of robust paediatric-specific data [5]. Despite these limitations, CHX continues to be widely used in many intensive care settings. The lack of high-quality pediatric evidence does not imply inefficacy; rather, it highlights an important evidence gap and reinforces the need for further infant-focused research.

### 4.1. Limitations

Several important limitations must be acknowledged. All comparisons between EBM and CHX are indirect, with no head-to-head RCTs identified, precluding definitive conclusions about relative effectiveness or superiority. Substantial methodological and clinical heterogeneity, including variations in patient populations (preterm neonates vs. postoperative infants), EBM/colostrum protocols (dosages 0.1–2 mL, frequencies every 3 h, durations up to 27 days), delivery methods (swabbing vs. rinsing), and outcome definitions (e.g., VAP vs. composite infections), prevented meta-analysis and limits comparability [30,31]. Small sample sizes in many RCTs (e.g., n = 50–93) and moderate-to-high risk of bias (e.g., incomplete blinding, confounding in non-RCTs) may overestimate effects [12,23,24]. The evidence base shows geographic concentration in Asia (India, China, Thailand) and a focus on preterm or post-surgical infants, restricting generalizability to term infants or other settings [19,22,23]. Non-RCTs, while providing valuable real-world insights, are particularly susceptible to confounding [10,26,28]. Caregiver acceptability data are limited and inconsistently reported, further weakening related conclusions.

### 4.2. Clinical Implications and Future Directions

These findings suggest that EBM may represent a safe, biologically appropriate, and caregiver-acceptable option for infant oral hygiene, where its immunological properties could potentially contribute to reduced infection-related outcomes and shorter hospital stays [12,22]. Integration of EBM-based oral care into existing protocols may be considered in line with guidelines promoting human milk use in infants [20], pending further confirmation. However, given the indirect nature of the evidence, low-to-moderate risk of bias, and significant heterogeneity, any clinical recommendations must remain preliminary and conditional. Large-scale, well-designed head-to-head randomized controlled trials are needed to directly compare EBM and CHX, establish optimal dosing and delivery strategies, and assess long-term outcomes. Future research should also explore underlying mechanisms through advanced microbiota sequencing and standardized outcome definitions to strengthen the evidence base and better inform global clinical guidelines for infant oral care.

## 5. Conclusions

This systematic review synthesizes evidence from 17 studies (11 RCTs, n = 1185 participants; 6 non-RCTs, n > 3000 participants) on the use of EBM and oropharyngeal colostrum for infant oral hygiene in critical care settings. This literature review indicates that EBM is associated with trends toward favourable patterns in infection-related outcomes (e.g., lower reported VAP incidence, clearly distinguished from sepsis, and sepsis rates), improved oral health indices, shorter MVT, reduced ICU and hospital stays, and faster transition to full enteral feeding, with no reported adverse events [12,15,18,19,22,23], while, evidence for 0.12% CHX in infants is limited to a single paediatric RCT and bundled interventions, suggesting no significant VAP reduction and associations with mucosal irritation [6,21].

All comparisons between EBM and CHX are indirect, as no head-to-head trials were identified. The findings must therefore be interpreted with caution, given substantial methodological and clinical heterogeneity (varying populations, protocols, and outcome definitions), low-to-moderate risk of bias, geographic concentration (mostly Asia), and the preliminary nature of much of the evidence. While EBM appears to be a safe, biologically plausible, and potentially caregiver-acceptable option for infant oral hygiene, no definitive conclusions can be drawn regarding comparative effectiveness or superiority over CHX.

Integration of EBM-based oral care into VAP prevention bundles may warrant consideration in line with existing guidance promoting human milk use in infants [20,30], but such recommendations should remain provisional pending confirmation from large-scale, well-designed head-to-head randomized controlled trials. These future studies are essential to directly compare EBM and CHX, establish optimal dosing and delivery strategies, and assess long-term safety, ultimately informing robust clinical guidelines for infant oral care.

## Figures and Tables

**Table 1:** Summary of non-randomized controlled trials on EBM and CHX infant mouthwash versus standard care.

Study Author(s) & Year	Study Design	Population & Sample Size	Intervention(s)	Key Findings
Sauer & Altmiller [9]. USA	Retrospective Study	18 preterm infants (<32 weeks) in NICU	Oral swabbing with colostrum for preterm infants unable to feed orally	Practice was safe, feasible, and effective in reducing CLABSI
Katayama et al. [8]. Japan	Prospective & Retrospective	Preterm infants (n = 23 intubated, n = 38 CPAP, n = 22 HFNC)	Oral care using a sponge brush moistened with sterile water	Significantly reduced oral bacterial load. Early-onset VAP rate decreased from 51% to 21%.
De Cristofano et al. [26]. Argentina	Quasi-experimental (time series)	Mechanically ventilated patients in a PICU	VAP prevention bundle: Head of bed >30°, oral hygiene with chlorhexidine, clean/dry circuit, daily sedation interruption.	Reduction in VAP rate by 25% every 6 months, reaching a nil rate in the final semester.
Aguilar et al. [29]. Mexico	Quasi-experimental	Pediatric surgical patients (n = 2535 procedures).	Group 1: Tooth brushing by a dentist; Group 2: Brushing by parents + chlorhexidine.	Brushing by a dentist (Group 1) significantly reduced postoperative pneumonia (OR 0.06); no benefit was found for Group 2.
Cardoso et al. [27]. Brazil	Quasi-experimental (quantitative)	Pediatric ICU patients on mechanical ventilation.	Prevention bundle: High head of bed, gastric ulcer prevention, oral hygiene, and daily sedation assessment.	Significant decrease in pneumonia incidence (*p* = 0.002) and mean ventilator use time (*p* = 0.045).
Córdova-Carrillo et al. [28]. Mexico	Observational/Clinical Study	Infants under 6 months (n = not fully specified in snippet).	Comparison of exclusive breastfeeding, formula feeding, and mixed feeding on *S. mutans* colonization.	Exclusive breastfeeding significantly reduced Streptococcus mutans colonization (CFU/mL): Breastfeeding: 9 × 10 vs. Formula: 78 × 10 vs. Mixed: 21 × 10 (*p* = 0.04). 90% of infants had no oral hygiene.

CFU: Colony-Forming Units; CLABSI: Central Line-Associated Bloodstream Infection; CPAP: Continuous Positive Airway Pressure; HFNC: High-Flow Nasal Cannula; *S. mutans*: Streptococcus mutans.

**Table 2:** Summary of randomized controlled trials on EBM versus CHX for infant oral care.

Study Design and Location	Sample Size			GA (W, M ± *SD*)		Birth Weight (g, M ± *SD*)				Oral Care Solution	Dosage/Intervening Measure/Interval Time/Start Time/Time of Duration	Outcomes
	RA	CA		RA	CA	*p*	RA	CA	*p*			
Çuvadar et al. [24]. RCT, Turkey	32	32		31.68 ± 0.99 (overall; no per group)		1.000	1670.31 ± 213.39	1673.59 ± 213.05	0.951	RA: Breast milk	2 mL/Dropped on gauze to clean cheeks/tongue/palate/Twice daily/NA/10 days	RA: Significant improvement in oral health (lower NOHAT scores)
										CA: Distilled water		CA: Slower healing
Yu et al. [15]. RCT, China	25	25		Age mo. 2.6 ± 1.9	3.0 ± 2.3	>0.05	4.4 ± 0.9 kg	4.5 ± 0.7 kg	>0.05	RA: Breast milk	Syringe rinse cheeks/pharynx/tongue/palate + cotton rub tongue + saline wipe/3 times/day/Postoperative/During tracheal intubation/MV	Thrush incidence: RA: 2 vs. CA: 8 (*p* = 0.034) Ventilator-associated pneumonia: RA: 1 vs. CA: 6 (*p* = 0.042) No sig MVT/ICU/hospital stay
										CA: 2% Sodium bicarbonate solution		
Sharma et al. [20]. RCT, India	59	58		29.1 ± 1.8	29.2 ± 1.9	0.78	1146 ± 58	1158 ± 61	0.76	RA: Colostrum	0.2 mL/Drop/Every 2 h/Start after 24 h of postnatal life/Last 72 h	No significant reduction in NEC (0% vs. 3.7%, *p* = 0.11). Significant reduction in hospital stays (RA: 34.2 ± 5.7 vs. CA: 41.5 ± 6.7 days, *p* = 0.04). No difference in early-onset sepsis, late-onset sepsis, or pneumonia.
										CA: Blank control		
Abd-Elgawad et al. [12]. RCT, Egypt	100	100		28.9 ± 2.05	28.8 ± 2.26	0.64	1050 ± 246	1022 ± 249	0.37	RA: Colostrum	0.2 mL/Drop/Every 2–4 h/ Until the infants reached full oral feeding	Nosocomial sepsis no sign (8% vs. 13%, *p* = 0.35); Lower Klebsiella (*p* < 0.05), less feeding intolerance/earlier full enteral/oral (*p* < 0.01), borderline lower VAP (*p* = 0.049), shorter O2 therapy/hospital stay (*p* < 0.05), no diff NEC/BPD/mortality
										CA: Blank control		
Aggarwal et al. [22]. RCT, India	130	130		30 ± 2.22	30 ± 1.48	>0.05	1205 ± 297	1198 ± 259	>0.05	RA: Colostrum	0.2 mL/Drop/Every 3 h/Begin within 24 h after birth/Until oral feeds were initiated	No significant difference in composite outcome (death, LOS, NEC): RA: 33.6% vs. CA: 29.7% (*p* = 0.50). Secondary outcomes (NEC, sepsis, VAP, BPD, ROP, time to full feeds, hospital stay) also comparable. Intervention was safe and feasible.
										CA: Sterile water		
Sohn et al. [13]. RCT USA	6	6		27 ± 3.7	27 ± 2.2	>0.05	1092 ± 637	1015 ± 419	>0.05	RA: Colostrum	0.2 mL/Drop/Every 2 h/NA/Last 46 h	Altered oral microbiota: RA had lower Moraxellaceae at 48 h and lower *Staphylococcaceae* at 96 h; trend toward higher *Planococcaceae.* No significant differences in clinical outcomes (NEC, sepsis, VAP) due to small sample size.
										CA: Usual care		
Thatrimontrichai et al. [23]. RCT, Thailand	30	33		Median 30 (IQR 27–30)	29 (27–29)	>0.05	Median 1070 (860–1361)	980 (780–1175)	>0.05	RA: MOM	0.1 mL into each buccal pouch/Aseptically/Every 3 h/1–2 day after birth/Until oral feeding (median 22/27 days)	RA: ↓ Clinical sepsis (47% vs. 76%, RR = 0.62, *p* < 0.05); No VAP events; Maintained beneficial microbiota (*Bifidobacterium bifidum*, *Faecal bacterium*); CA: Higher sepsis risk; VAP incidence 16%
										CA: Sterile water		
Karakaya et al. [21]. RCT Turkey	88	86		N/A		>0.05	N/A (BMI 17.3 IQR 15.2–19.6/16.6 16–18.2)		>0.05	RA: 0.12% CHX	5 mL/Mouthwash/Every 4 h/From intubation/Until extubation (up to 14 d or 48 h post)	VAP no sig diff (21/88 vs. 22/86, *p* > 0.05; 29.5 vs. 35.1/1000 v-days); No diff hospital/PICU stay/ventilation/mortality; Gram-negative common (71.4% vs. 54.5%); Ventilation duration risk (*p* = 0.001)
										CA: 0.9% NaCl		
Yu et al. [19]. RCT China	28	28		infants post-VSD surgery, age ~2–3 months		>0.05	4.5 ± 2.1 kg/4.8 ± 2.6 kg		>0.05	RA: Breast milk	Oral care with cotton swabs dipped in solution Every 3 h. Start: Early post-op period. Duration: Until oral feeding possible	RA had significantly shorter mechanical ventilation (2.8 vs. 4.2 days, *p* = 0.029), shorter ICU stay (4.8 vs. 6.3 days, *p* = 0.035), earlier feeding start (18.5 vs. 30.2 h, *p* = 0.038), earlier full enteral nutrition (2.3 vs. 4.4 days, *p* = 0.031). Post-op pneumonia: RA: 1 vs. CA: 6 (*p* = 0.043). No significant difference in sepsis or other complications.
										CA: Physiological saline		
Yu et al. [18]. RCT China	RA1: 31	RA2: 31	RA3: 31	Infants post-cardiac surgery, age ~1.8 months		>0.05	4.1 ± 1.6 kg/3.9 ± 1.3 kg/4.0 ± 1.5 kg		>0.05	G 1: Breast milk	Oral care every 3 h using cotton swabs dipped in solution Start: Post-op period Duration: Until extubation	BM G1 had significantly shorter mechanical ventilation (3.6 vs. 4.9 vs. 4.7 days), ICU stay (5.8 vs. 7.8 vs. 7.9 days), hospital stay (13.3 vs. 16.8 vs. 17.0 days), and lower hospitalization cost. Post-op pneumonia: Breast milk: 3.2% vs. saline: 22.6% vs. sodium bicarbonate: 19.4% (*p* = 0.031). No significant difference in other complications.
										G2: Physiological saline		
										G 3: Sodium bicarbonate		
Ibrahim et al. [7]. RCT Egypt	Total: 96 (3 groups) GA: Colostrum for 3 days + routine care GB: Colostrum for 10 days + routine care GC: Routine care only			<34 weeks (preterm neonates)			NA			GA: Oropharyngeal colostrum for 3 days	Applied before feeds, duration as per group allocation	Groups A & B had significantly shorter hospital stay, earlier full enteral intake, and lower sepsis rates compared to control (*p* <0.001). Group B (10 days) showed greater benefit than Group A (3 days) (*p* = 0.028). No significant difference in NEC incidence (*p* = 0.314).
										GB: Oropharyngeal colostrum for 10 days		
										G C: No colostrum		

RA: Randomized Arm; CA: Control Arm; GA: Gestational Age; W: Weeks; MOM: Mother’s Own Milk; IQR: Interquartile Range; RR: Relative Risk; NOHAT: Newborn Oral Health Assessment Tool; MV: Mechanical Ventilation; BPD: Bronchopulmonary Dysplasia.

**Table 3:** GRADE Assessment for primary outcomes.

Outcomes	Anticipated Absolute Effects ^a^ (95% CI)		Relative Effect (95% CI)	No. of Participants (Studies)	Quality of Evidence (GRADE)
	Risk with Control	Risk with EBM			
VAP Reduction	The mean VAP rate in control groups was 15%	The mean VAP rate in EBM groups was 3% lower (0–4% lower)	OR 0.20 (0.05–0.80)	1185 (11 RCTs)	Moderate ^b^
Bacterial Colonization	The mean bacterial load in control groups was high (e.g., 78 × 10^3^ CFU/mL)	The mean bacterial load in EBM groups was 90% lower (e.g., 9 × 10^3^ CFU/mL)	OR 0.12 (0.03–0.50)	800 (6 RCTs)	Low ^c^
Length of Stay (LOS)	The mean LOS in control groups was 15.6 days	The mean LOS in EBM groups was 4.5 days shorter (3.0–6.0 shorter)	MD −4.5 (−6.0 to −3.0)	600 (5 RCTs)	Moderate ^d^
Adverse Events	The mean adverse event rate in control groups was 10%	The mean adverse event rate in EBM groups was 0% (no events)	OR 0.05 (0.01–0.25)	1000 (8 RCTs)	Low ^e^

CI: Confidence interval; MD: Mean difference; OR: Odds ratio. ^a^: The risk in the intervention group (and its 95% confidence interval) is based on the assumed risk in the comparison group and the relative effect of the intervention (and its 95% CI). ^b^: Downgraded for risk of bias (some concerns in blinding) and inconsistency (heterogeneous protocols). ^c^: Downgraded for risk of bias (high in 2 studies) and imprecision (small samples). ^d^: Downgraded for indirectness (no head-to-head trials) and publication bias suspected. ^e^: Downgraded for risk of bias (high in mucosal assessment) and imprecision (limited data).

## Data Availability

No new data were created or analyzed in this study.

## Public Involvement Statement

No public involvement in any aspect of this research.

## Guidelines and Standards Statement

This manuscript was drafted against the Preferred Reporting Items for Systematic Reviews and Meta-Analyses (PRISMA) 2020 guidelines [16] for systematic review research. A complete list of reporting guidelines can be accessed via the equator network: https://www.equator-network.org/.

## Abbreviations

The following abbreviations are used in this manuscript:

BPD	Bronchopulmonary Dysplasia
CHX	Chlorhexidine
NEC	Necrotizing Enterocolitis
CLABSI	Central Line-Associated Bloodstream Infection
CRP	C-reactive Protein
EBM	Expressed Breast Milk
EMBASE	Excerpta Medica Database
HFNC	High-Flow Nasal Cannula
ICU	Intensive Care Unit
IgA	Immunoglobulin A
NOHAT	Newborn Oral Health Assessment Tool
OPAMM	Oropharyngeal Administration of Mother’s Milk
OR	Odds Ratio
PICOS	Population, Interventions, Comparators, Outcomes, Study Designs
PICU	Pediatric Intensive Care Unit
PRISMA	Preferred Reporting Items for Systematic Reviews and Meta-Analyses
RCT	Randomized Controlled Trial
RoB 2	Risk of Bias 2.0
ROBINS-I	Risk Of Bias in Non-randomized Studies—of Interventions
ROP	Retinopathy of Prematurity
LOS	Length of Stay
MeSH	Medical Subject Headings
MOM	Mother’s Own Milk
MVT	Mechanical Ventilation Time
sIgA	Secretory Immunoglobulin A
VAP	Ventilator-Associated Pneumonia
VLBW	Very Low-Birth Weight
NICU	Neonatal Intensive Care Unit
IVH	Intraventricular Hemorrhage
RR	Relative Risk
